# Assessing the proximate compositions of indigenous forage species in Yemen’s pastoral rangelands

**DOI:** 10.1515/biol-2022-0901

**Published:** 2024-11-11

**Authors:** Mounir Louhaichi, Basel Abdulla Salem Al-koor, Mouldi Gamoun, Anwar Adam Abdulgader Abdurahman, Sawsan Hassan

**Affiliations:** International Center for Agricultural Research in the Dry Areas (ICARDA), Tunis, 1004, Tunisia; Department of Animal and Rangeland Science, Oregon State University, Corvallis, OR, 97331, United States of America; Agricultural Research and Extension Authority (AREA), Dhamar, 87148, Yemen; Higher Institute of Arts and Crafts of Tataouine (ISAMT), Mahrajène City, BP 47, Tataouine, Tunisia

**Keywords:** climate changes, forage palatability, indigenous species, livestock grazing

## Abstract

Plant diversity in southern Yemen is crucial for maintaining rangeland ecosystem functions. This diversity contributes to the resilience of local pastoral communities, by providing essential forage and resources. However, high stocking density has led to the overuse of palatable species, resulting in increased competition for forage. This study evaluates the nutritional value of 25 indigenous forage species from the natural rangelands of Lahij Governorate. Significant variations were observed among the Forage species, with moisture content ranging from 4 to 39.6%, crude protein from 5.5 to 21.4%, non-fiber carbohydrates from 31.8 to 66.4%, crude fiber from 8.3 to 42.65%, and ash content from 9.2 to 34.6%. *Clitoria ternatea*, *Lycium barbarum*, *Senegalia mellifera*, *Vigna sinensis*, *Albizia lebbeck*, and *Acacia trees* with crude protein content higher than 16% showed substantial potential as livestock feed due to their favorable proximate compositions. Incorporating these high-potential species into regular livestock diets could significantly enhance the sustainability and productivity of pastoral systems in southern Yemen, addressing the current fodder shortage.

## 1 Introduction

Yemen boasts a remarkable and diverse array of plant species, nurtured by its unique ecological landscape. This plant diversity is a reflection of the country’s varied climatic and edaphic conditions. Yemen’s biodiversity holds great significance as it profoundly impacts the nation’s ecosystems, economy, and culture. It plays a pivotal role in sustaining Yemen’s delicate ecosystems, providing vital resources for livelihoods, preserving traditional knowledge and cultural heritage, and offering an array of ecosystem services. Numerous researchers have explored Yemen’s biodiversity, revealing six distinct ecological zones within the country [1,2,3,4,5,6,7,8,9]. These ecological zones include (1) Coastal Plains Regions (Tihama), where an impressive 264 wild plant species have been documented. The western coastal areas exhibit diverse vegetation, including mangroves like *Avicennia marina* (Forssk.) Vierh. and halophytes such as *Salsola spinescens* Moq., *Suaeda monoica* Forssk. ex J.F.Gmel., *Atriplex* sp., and *Aeluropus lagopoides* (L.) Thwaites. Transitioning to the flat sandy plains of the coastal plain’s regions, various plant communities emerge, featuring species like *Senna*, *Aerva*, *Dipterygium*, and *Blepharis*. As one moves toward the foothills of Tihama, the vegetation transforms, marked by the presence of plants like *Dobera glabra* Juss. ex Poir., various *Acacia* species, *Adenium obesum* (Forssk.) Roem. & Schult, and a diverse array of *Euphorbia* species. (2) The Southern Coastal Plain, which hosts halophytes, including species like *Salsola vermiculata* L., and *Tamarix nilotica* (Ehrenb.) Bunge. among others. This region also features thriving vegetation on flat sandy plains and sand dunes, with species like *Crotalaria microphylla* Vahl, *Fagonia indica* L., and *Gloriosa revoilii* (Franch.) J.C. Manning & Vinn. Notable additions to this diverse ecosystem include *Pluchea indica* subsp. and *Euphorbia dracunculoides* Lam. (3) The Mountainous Regions showcase a diverse landscape. In low-altitude mountains, a mix of subshrubs, shrubs, and dwarf trees like *Dobera glabra* (Forssk.) Juss. ex Poir., *Acacia* species, *Commiphora myrrha* (T.Nees) Engl., *Grewia* spp., and *Crotalaria saltiana* Andrews can be found. As one ascends to medium altitude mountains, the scenery changes to include shrubs and trees like *Tamarindus indica* L., *Combretum molle* R. Br ex G. Don., *Grewia schweinfurthii* Burret, and *Olea europaea* L. High altitude mountains are particularly rich in subshrubs, herbs, and ferns, featuring species such as *Rosa abyssinica* Lindl., *Otostegia fruticosa* Forssk., *Salvia aegyptiaca* L., and other unique varieties. (4) The highland plains are characterized by the presence of *Acacia*, *Jatropha*, *Brassica tournefortii* Gouan., *Hibiscus*, and other species, both perennial and annual, contributing to the region’s natural vegetation alongside cultivated crops. (5) In the Eastern Desert, there is limited floristic diversity, with sparse shrub communities such as *Calligonum comosum* L’Hér. and *Haloxylon persicum* Bunge. ex Boiss. & Buhse. Sand dunes are inhabited by *Leptadenia pyrotechnica* (Forssk.) Decne., *Salvadora persica* L., and *Panicum turgidum* Forssk. Some shrubs and subshrubs grow in shallow wadis. (6) The islands in the Red Sea and Indian Ocean closely resemble the Western Coastal Plain in terms of vegetation. These islands house mangroves, halophytes, and various xerophytic plants. Notably, some islands, particularly those in the Socotra Archipelago, boast unique endemics like *Dracaena cinnabari* Balf.f., *Commiphora socotrana* (Balf.f.) Engl., and *Euphorbia socotrana* Balf.f.

Yemen’s extensive rangelands cover approximately 40% of its total land area and are essential for the nation’s economy and food security [10,11]. These vast expanses are not merely geographical features; they are essential sources of livelihood for the Yemeni people, providing crucial sustenance and income through livestock husbandry, including sheep, goats, and camels. These animals are a means of livelihood and the very essence of life for many Yemeni households [12,13].

Yemen’s rangelands encompass a wide range of climatic conditions, ranging from arid deserts to rugged mountainous terrains and coastal areas [12]. This rangeland diversity includes a rich natural forage plant community, mirroring the nation’s varied landscapes and climates. These plants are highly valued for their high nutritional content and remarkable drought resistance, making them essential for sustaining Yemen’s livestock industry [14,15]. Beyond their role in supporting local livestock, these remarkable species have evolved unique adaptations, enabling them to thrive in Yemen’s harsh arid and semi-arid climates and provide crucial ecosystem services. They offer shade, mitigate soil erosion, and serve as habitats for wildlife species. The preservation and propagation of these natural fodder species are of high significance, not only in terms of improving livelihoods and food security but also in bolstering the resilience of Yemeni ecosystems [16].

The nutritional value of Yemen’s indigenous forage species encompasses a wide spectrum of diversity, with certain plants emerging as veritable nutritional powerhouses for livestock. Indigenous forage species like *P. turgidum* Forssk. and *Atriplex halimus* L., as highlighted by Attia-Ismail [17], exhibit rich profiles in terms of protein and minerals, rendering them invaluable resources for animal nutrition. Nevertheless, the full realization of the potential of these forage resources hinges on further research aimed at identifying and optimizing their use to enhance livestock productivity and sustainability.

Exploring the chemical composition of indigenous forage species, including the grasses and legumes thriving in Yemen’s rangelands, is instrumental in understanding their role in the nutrition and well-being of various herbivorous animals [18]. These indigenous forage species contain a diverse array of organic compounds, with their nutritional value varying between species. Generally, rangeland forages are rich in carbohydrates, crude protein, crude fat, and crude fiber [19]. Carbohydrates serve as the primary energy source for livestock [20], while fiber plays an important role in supporting rumen function and microbial activity [21]. Additionally, forage species have varying levels of crude proteins, which are indispensable for animal performance [22]. These forages are also replete with essential vitamins and minerals, including calcium and phosphorus, which are critical for overall animal health and metabolic function [23]. The presence of secondary compounds, such as tannins and alkaloids, further contributes to the complexity of forage species, influencing palatability and, at times, exerting positive or negative effects on animal health [24].

However, rangelands face significant challenges that pose a substantial threat to sustainability. One such challenge is the excessive grazing pressure especially on palatable species, leading to a decline in available forage and subsequent impacts on livestock production. The high grazing intensity of palatable species, driven by increasing livestock numbers has resulted in the degradation of rangelands [25]. This degradation reduces the productivity and diversity of plant species essential for livestock nutrition. As a result, the reduction in preferred, highly nutritious plant species that livestock graze on can lead to an increase in the presence of unpalatable species [26], compelling livestock to consume these less nutritious plant species, ultimately resulting in lower livestock health, reduced reproduction rates, and decreased milk and meat production [27]. In Yemen, the challenges of deforestation and degradation, arising from factors like conflict and prolonged droughts, are aggravating these challenges. These challenges significantly contribute to soil erosion, loss of biodiversity, and heightened vulnerability to climate change, further complicating the plight of pastoral communities. Recent years have witnessed a growing demand for fodder resources, driven by various pressing factors, including diminishing rainfall, mismanagement in arid areas, and the additional challenge of political instability [28,29]. These factors collectively affect the availability and accessibility of fodder, as disruptions caused by political conflicts can impede the distribution and management of rangeland resources, further exacerbating the strain on pastoral communities and their livestock. Studies conducted by Al-Hawshabi and El-Naggar [7] have documented numerous endemic and native species in Yemen, shedding light on the ecological significance and untapped economic potential hidden within these natural rangelands. This increased demand has had a significant impact on Yemen’s livestock sector, affecting the number of livestock and price fluctuations.

In light of these challenges, the primary objective of this study was to conduct a comprehensive assessment of the proximate composition of 25 prominent indigenous forage species frequently used for animal grazing in the Lahij rangelands. This assessment was undertaken to clarify their nutritional content, with the aim of determining their suitability for addressing Yemen’s feed-related challenges. This, in turn, could lead to improved livestock health, productivity, and overall resilience. Furthermore, understanding the nutritional composition of these indigenous forage species can inform the decision-makers of more effective rangeland management strategies.

## 2 Materials and methods

### 2.1 Study area

**Figure 1:**
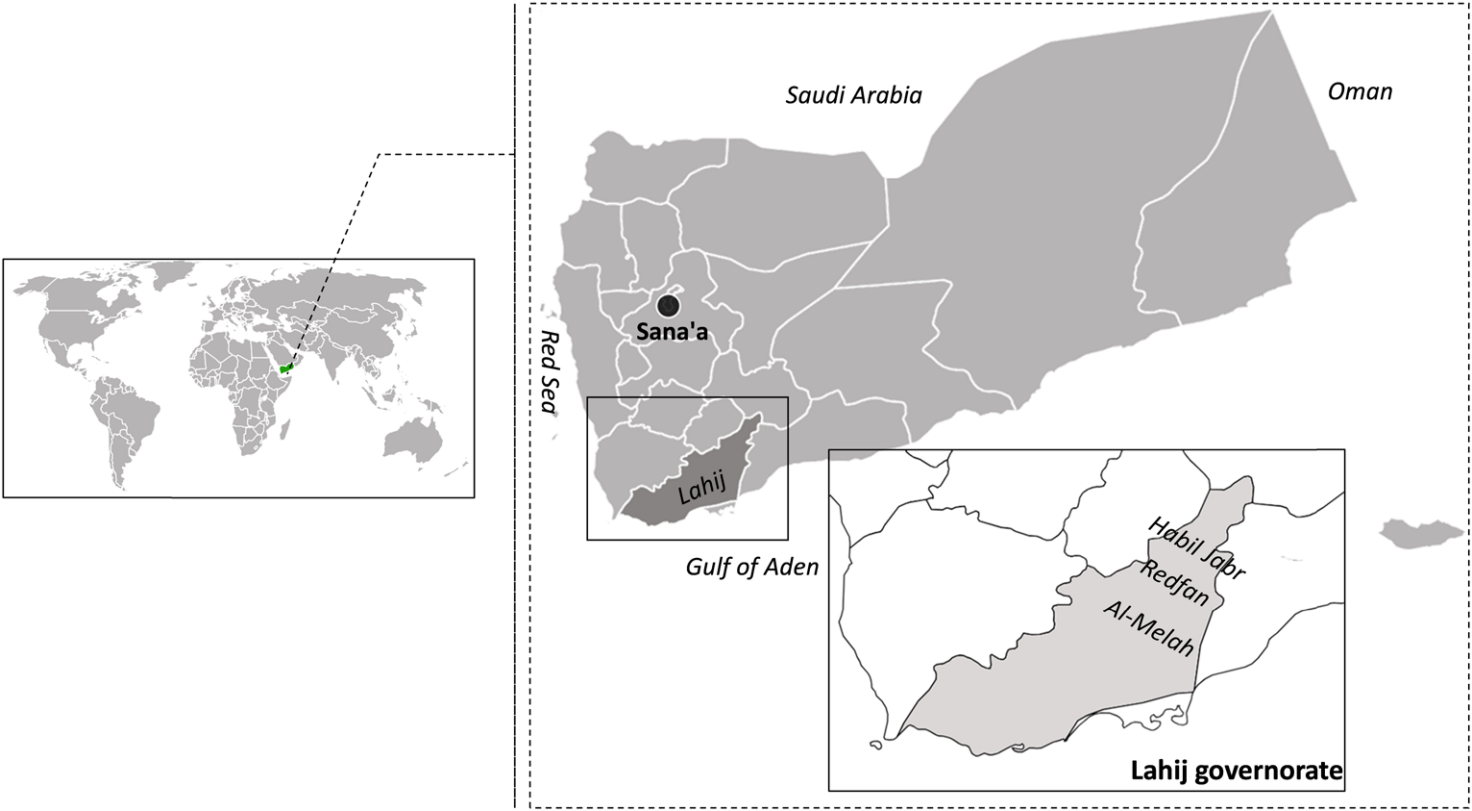
Map of Yemen study sites in Habil Jabr, Redfan, and Al-Melah, Governorate of Lahij, Southern Yemen.

The study area is located in the Lahij Governorate on the southwest coast of the Republic of Yemen (Figure 1). Lahij, situated in a subtropical arid region, is known for its scorching summers and mild winters. Summer temperatures along the coastal plains can reach up to 32°C, while winter temperatures average around 20°C [30]. Rainfall occurs during the winter and autumn months. Rangelands of Lahij are diverse, rising to elevations of up to 2,500 m above sea level. These lands are characterized by high biodiversity, with the legume family being one of the most important and widespread families in the region, comprising a total of 63 taxa. In 2022, various rangeland locations across three districts in the Lahij region – Al-Melah, Redfan, and Habil Jabr – were selected for sampling 25 indigenous fodder species. These locations, situated in the northern part of Lahij, predominantly feature shallow soils, with the deepest soils found in terraced slopes and valleys.

### 2.2 Species collection

Twenty-five indigenous forage species, including 2 trees, 15 shrubs, and 8 herbaceous species commonly utilized for animal grazing in the Lahij rangelands, were chosen for evaluation. These species were selected based on information gathered through direct surveys conducted with local pastoralists, aimed at identifying plants with significant grazing value for livestock, particularly small ruminants. These species are distributed across ten plant families: Amaranthaceae, Cleomaceae, Fabaceae, Malvaceae, Poaceae, Polygonaceae, Salvadoraceae, Scrophulariaceae, Solanaceae, and Zygophyllaceae (Table 1). The palatability index covers a range from 0 to 5, representing different levels of palatability: highly palatable (5), very palatable (4), palatable (3), fairly palatable (2), occasionally palatable or poorly palatable (1), and not palatable (0) [30]. In 2022, during the full bloom stage of their growth cycle, three plants per species with similar size and age were carefully selected. From each forage tree species, we collected 1 kg of edible biomass. Shrubs were pruned to one-third of their above-ground height, isolating the grazable materials from the woody parts (leaves and stems less than 10 mm in diameter). Herbs were uniformly harvested, leaving a 15 cm above-ground stubble. We then combined the samples of each species into a single composite sample for analysis.

**Table 1:** Family, habitat class, and grazing acceptability of studied indigenous forage species of southern Yemen

Species	Family	Habitat class	Palatability index*
*Abutilon bidentatum* (Hochst) A. Rich.	Malvaceae	Shrub	5
*Acacia etbaica* (Schweinf.) Kyal. & Boatwr.	Fabaceae	Tree	5
*Acacia hamulosa* Benth.	Fabaceae	Shrub	4
*Aerva javanica* (Burm. f.) Juss. ex Schult.	Amaranthaceae	Shrub	3
*Albizia lebbeck* (L.) Benth	Fabaceae	Tree	4
*Amaranthus* spp.	Amaranthaceae	Shrub	5
*Anticharis arabica* Endl.	Scrophulariaceae	Shrub	5
*Cenchrus ciliaris* L.	Poaceae	Herb	5
*Clitoria ternatea* L.	Fabaceae	Shrub	5
*Cynodon dactylon* (L.) Pers.	Poaceae	Herb	5
*Dipterygium glaucum* Decne.	Cleomaceae	Shrub	5
*Fagonia indica* Burm.f	Zygophyllaceae	Shrub	5
*Indigofera oblongifolia* Forssk.	Fabaceae	Shrub	4
*Indigofera spinosa* Forssk.	Fabaceae	Shrub	4
*Lycium barbarum* L.	Solanaceae	Shrub	4
*Ornithopus sativus* Brot.	Fabaceae	Shrub	4
*Panicum antidotale* Retz.	Poaceae	Herb	4
*Panicum turgidum* Forssk.	Poaceae	Herb	4
*Pennisetum purpureum* Schumach.	Poaceae	Herb	5
*Rumex nervosus* Vahl	Polygonaceae	Shrub	3
*Salvadora persica* L.	Salvadoraceae	Shrub	3
*Senegalia mellifera* (Benth.) Seigler & Ebinger	Fabaceae	Shrub	4
*Stipagrostis ciliata* (Desf.) De Winter	Poaceae	Herb	5
*Tribulus terrestris* L.	Zygophyllaceae	Herb	3
*Vigna sinensis* (L.) Savi ex Hassk.	Fabaceae	Herb	4

*Palatability index: 0, not palatable; 1, occasionally palatable; 2, fairly palatable; 3, palatable; 4, very palatable; 5, highly palatable.

### 2.3 Samples processing and analysis

Various parameters contribute to defining forage quality, reflecting the nutritional value of feeds and the presence and accessibility of essential nutrients, as demonstrated by Olowu and Yaman Firincioğlu [31]. Determining these parameters involves chemical and physical analyses, with additional crucial parameters that can be calculated based on laboratory results [32]. Understanding these parameters is imperative since they directly affect the nutritional value and digestibility of forage.

In this study, moisture content (%) was determined using the standard oven-drying method [33]. Approximately 5 g of each sample were dried at 105°C until a constant weight was achieved. Moisture content (%) was calculated using the equation:

\[\text{Moisture}\hspace{.5em}\text{content}( \% )={[}(\text{Initial}\hspace{.5em}\text{weight}-\text{Final}\hspace{.5em}\text{weight})/\text{Initial}\hspace{.5em}\text{weight}]\times 100.]\]

The remaining samples were oven-dried at 60°C for 48 h and then ground in a Thomas Model 4 Wiley mill, passing through a 1 mm sieve before analysis. Our nutritional component analysis followed established methods. Crude protein content (CP%) was assessed using the Kjeldahl method [34], where 0.5 g of each sample was digested, and ammonia released during digestion was titrated. Crude fat or ether extracts (%) were determined by employing the Soxhlet extraction method [35], with 2 g of each sample extracted using petroleum ether. Ash content (%) was estimated by incineration at 600°C [36] using 2 grams of each sample. Non-fiber carbohydrate content (NFC%) was calculated by subtracting the sum of crude protein, crude fat, ash, and crude fiber percentages from 100% [37]. Crude fiber content (%) analysis involved homogenizing and grinding the samples into particles smaller than 0.5 mm. The determination of crude fiber content was conducted by dissolving the samples in a solution containing 0.255 N H_2_SO_4_ and 0.13 N NaOH, following the guidelines outlined in Le Houérou and Ionesco [38].

### 2.4 Data analysis

A completely randomized design utilizing individual plants as replicates was employed for the study. Data were subjected to statistical analysis using the general linear model in SAS software to assess variations. Significance levels were set at *P* < 0.05 to determine the significance of the observed effects. Cluster analysis, employing the average linkage method, was utilized to identify species clusters based on quality determinants and the palatability index.

## 3 Results and discussion

To rehabilitate degraded rangelands and promote indigenous forage species for potential animal feed, herbivores need a diverse dietary range, including herbaceous legumes, non-legumes, shrubs, and trees as their forage sources [39]. Analyzing the chemical composition of forage samples is crucial for determining their quality and ensuring optimal nutrition for livestock. The proximate analysis conducted on these 25 indigenous forage species revealed notable variations (Table 2). Fresh plant samples displayed a wide moisture range, from 4% in *Aerva javanica* to 39% in *Senegalia mellifera*. The moisture content in forage is a critical determinant affecting its storability, susceptibility to microbial growth, and nutritional value. Prior research has shown reducing moisture levels inhibits microbial proliferation, enhancing the forage’s storage potential [40]. Conversely, excessive moisture levels can foster significant anaerobic bacterial growth, including *Clostridium* species, that are affecting forage preservation [41]. As suggested by Van Soest et al. [42], forage with a moisture content below 15% is generally considered suitable for animal feed. Additionally, assessing crude protein content is vital when evaluating forage quality. Significant variations in crude protein levels were observed among the species (*P* < 0.001). *P. turgidum* exhibited the lowest content at 5.5%, while *Clitoria ternatea* displayed higher crude protein content at 21.4%. Furthermore, four other species demonstrated elevated levels of crude protein content: *S. mellifera* (18.4%), *Ornithopus sativus* (18%), *Vigna sinensis* (17.2%), and *Acacia etbaica* (17%) (Table 2). Leguminous species exhibited higher crude protein content, ranging from 9.2 to 21.4%, with an average of 15.6%. In contrast, grasses exhibited a range of 5.5–10.5%, with an average of 8.9%, falling within the range reported by Lee et al. [55]. In this study, trees recorded the highest protein content, followed by shrubs, while herbs exhibited the lowest crude protein values. This differentiation can be attributed to the selected tree species in this study being legumes, whereas the herbs primarily consist of grasses. Leguminous plant species exhibit elevated protein levels compared to grasses, primarily due to their proficiency in biological nitrogen fixation. This unique capacity enhances nitrogen uptake, thereby increasing protein content and positively influencing forage crude protein levels, as substantiated by prior research [43,44]. Besides, legumes possess distinctive leaf structures characterized by reduced cell wall content, a lower hemicellulose-to-cellulose ratio, and a higher lignin content within the cell wall, differentiating them from grass species [45]. Protein is essential for the growth, immune function, enzyme activity, hormone regulation, nutrient transport, and overall productivity of ruminants [46,47,48,49]. Higher protein content contributes to improved feed digestibility, enhancing animal performance [22]. Superior forage quality is typically associated with increased crude protein levels, with forages containing over 13% crude protein considered high quality and can serve as a supplement to poor-quality livestock feed [50]. Ruminants’ crude protein consumption in the diet varies from 7 to 20%, depending on the species, sex, and physiological condition while a minimum of approximately 3.6% crude protein in feed is mandatory [51,52]. The crude protein content of these indigenous forage species closely resembles that of other species found in the arid rangelands of Tunisia, such as *Rhanterium suaveolens* (13.51%), *Helianthemum lippii* (12.60%), *Echiochilon fruticosum* (12.31%), *Argyrolobium uniflorum* (11.08%), *Helianthemum kahiricum* (10.70%), *Gymnocarpos decander* (10.04%), *Anthyllis henoniana* (9.82%), *Plantago albicans* (8.85%), *Stipagrostis plumosa* (6.69), *Stipa lagascae* (6.06%), *Stipagrostis pungens* (5.92%), and *Stipagrostis tenacissima* (4.98%) [53]. Despite leguminous trees exhibiting the highest protein content, the availability of this nitrogen is influenced by the presence of secondary compounds, such as condensed tannins. These tannins have been identified in the *Acacia* genus [54] and *Albizia lebbeck* [55]. These secondary compounds play a significant role in affecting the degradation of protein within the rumen microbial ecosystem, subsequently impacting the availability of nitrogen in fodder species [56]. Additionally, changes in forage quality over time are closely linked to prevailing climatic conditions. During periods of drought, the reduction in nitrogen absorption capacity may exceed the decrease in plant growth, resulting in decreased nitrogen levels in the leaves [57]. This phenomenon can be attributed to temperature-related effects that favor tree and shrub species, directing their energy toward structural support and defense mechanisms rather than leaf growth, as demonstrated in previous research [58].

**Table 2:** Proximate analysis of 25 selected indigenous forage species from southern Yemen

Species	Moisture (%)	Crude protein (%)	Crude fat (%)	Ash (%)	Carbohydrate (%)	Crude fiber (%)
*Abutilon bidentatum*	15	10.2	2.4	15.1	54.3	18
*Acacia etbaica*	9	17	2.1	16.2	36.6	28.1
*Acacia hamulosa*	17.9	15.1	1.3	17.1	42.3	24.2
*Aerva javanica*	4	10	4.5	15.4	31.8	38.3
*Albizia lebbeck*	14.6	16.4	4.9	12.7	39.1	26.9
*Amaranthus* spp.	5.8	16.6	1.8	12	55.6	14
*Anticharis arabica*	11.2	9.5	2.8	27.2	43.6	16.9
*Cenchrus ciliaris*	12.6	9.2	2.1	15.1	33.35	40.25
*Clitoria ternatea*	16.3	21.4	3.3	9.3	40.1	25.9
*Cynodon dactylon*	14.7	9.6	1.8	14.7	42.4	31.5
*Dipterygium glaucum*	9.2	6	6.85	19.2	41.75	26.2
*Fagonia indica*	6	9	6.2	17	37.8	30
*Indigofera oblongifolia*	17.5	8.5	2.3	12.5	66.4	10.3
*Indigofera spinosa*	4.4	9.2	3.6	18	51.2	18
*Lycium barbarum*	10	13.8	2.5	14.5	48.3	20.9
*Ornithopus sativus*	5	18	4	14	44	20
*Panicum antidotale*	16.1	10.5	1.6	10.1	41	36.8
*Panicum turgidum*	4.8	5.5	2.9	9.8	39.15	42.65
*Pennisetum purpureum*	19.8	9.3	2	11.8	46.3	30.6
*Rumex nervosus*	8.4	13.2	1.4	18.9	58.2	8.3
*Salvadora persica*	31.3	16.3	2.2	34.6	35.8	11.1
*Senegalia mellifera*	39.6	18.4	2.5	9.2	45.9	24
*Stipagrostis ciliata*	13.8	9.2	1.4	12.8	48.58	28.02
*Tribulus terrestris*	14.3	16.2	2.9	13.3	51.4	16.2
*Vigna sinensis*	12.5	17.2	2.8	13.5	40.9	25.6
LSD (*P* < 0.05)	0.529	0.616	0.473	0.493	0.483	0.454
*P* < *0.05*	*<0.001*	*<0.001*	*<0.001*	*<0.001*	*<0.001*	*<0.001*

In this study, we found that crude fat levels varied from 1.3 to 6.85%. *Dipterygium glaucum* exhibited the highest fat content, while *Acacia hamulosa* displayed the lowest (Table 2). Crude fat analysis encompasses a diverse range of lipid compounds, including triglycerides, alcohols, waxes, terpenes, steroids, pigments, esters, aldehydes, and various other lipids [59]. In animal nutrition, crude fat serves as a vital component, providing a concentrated source of energy for livestock and contributing to the overall nutritional balance of their diets [47]. In some previous studies, it was found that the crude fat requirement is typically 2–5% for ruminants [60]. However, certain forage species, such as *Medicago sativa* and *Festuca kashmiriana*, have good forage potential, although they contain a low percentage of fat [61].

The crude fiber content among the 25 evaluated species showed significant variation, ranging from 8.3 to 42.7%. *P. turgidum* exhibited the highest crude fiber percentage while *Rumex nervosus* had the lowest. Fiber helps regulate the movement of food through the digestive system. High-fiber forage enhances digestive health by accelerating food passage, which can lead to reduced nutrient absorption and utilization rates, and ultimately a decline in feed efficiency. On the other hand, insufficient fiber can cause digestive issues such as constipation or diarrhea [62]. In this study, the fiber content of the species ranged from moderate to high [47] which may lower the digestibility and energy from fodder [63].

The non-fiber carbohydrate content (NFC%) of the experimental species ranged from 31.8% in *A. javanica* to 66.4% in *Indigofera oblongifolia*. These differences were statistically significant (*p* < 0.05), indicating a significant variation in the energy-providing components of the forage. An increase in NFC% content tends to increase its digestibility, due to their high digestibility. A lower NFC% content can enhance ruminant production efficiency. However, high NFC% feed has the potential to supplement rumen-degradable protein, reducing nitrogen losses. Therefore, balancing the ratio of energy to protein is crucial for maximizing the efficiency of the livestock production system while minimizing environmental impact [64]. In our study, only three species – *P. turgidum*, *D. glaucum*, and *I. oblongifolia* – showed high NFC% content compared to protein content, suggesting that most of the species included in this study have a good protein profile with less environmental impact.

Ash content serves as a parameter for assessing mineral composition in feed [65]. Higher ash content is frequently indicative of a greater mineral concentration. Although minerals are vital for animal health [66], excessive mineral content can lead to a reduction in the nutritional quality of the feed [67].

The analysis of ash content revealed values ranging from 1.1 to 44.8%. Notably, *S. persica* exhibited the highest ash content at 34.6%, while *C. ternatea* and *S. mellifera* displayed the lowest at 9.3 and 9.2% respectively (Table 2). The ash content of all forage species fell within the medium to high range (>5%). However, it is essential to note that, for specific species in arid Tunisian rangelands, the ash content ranged from 1 to 12%, as reported by Louhaichi et al. [22].

The palatability index reveals that 44% of the species considered are highly palatable, 40% exhibit good palatability, and the remaining 16% display moderate palatability. Notably, species like *R. nervosus*, *S. persica*, and *Tribulus terrestris* fall into the category of moderate palatability. These species are rich in secondary compounds, which are known to reduce the palatability and voluntary feed intake of forages [56,68]. However, these compounds are significant for mitigating methane emissions from ruminants, which have a significant environmental impact and play a role in addressing climate change [68].

**Figure 2:**
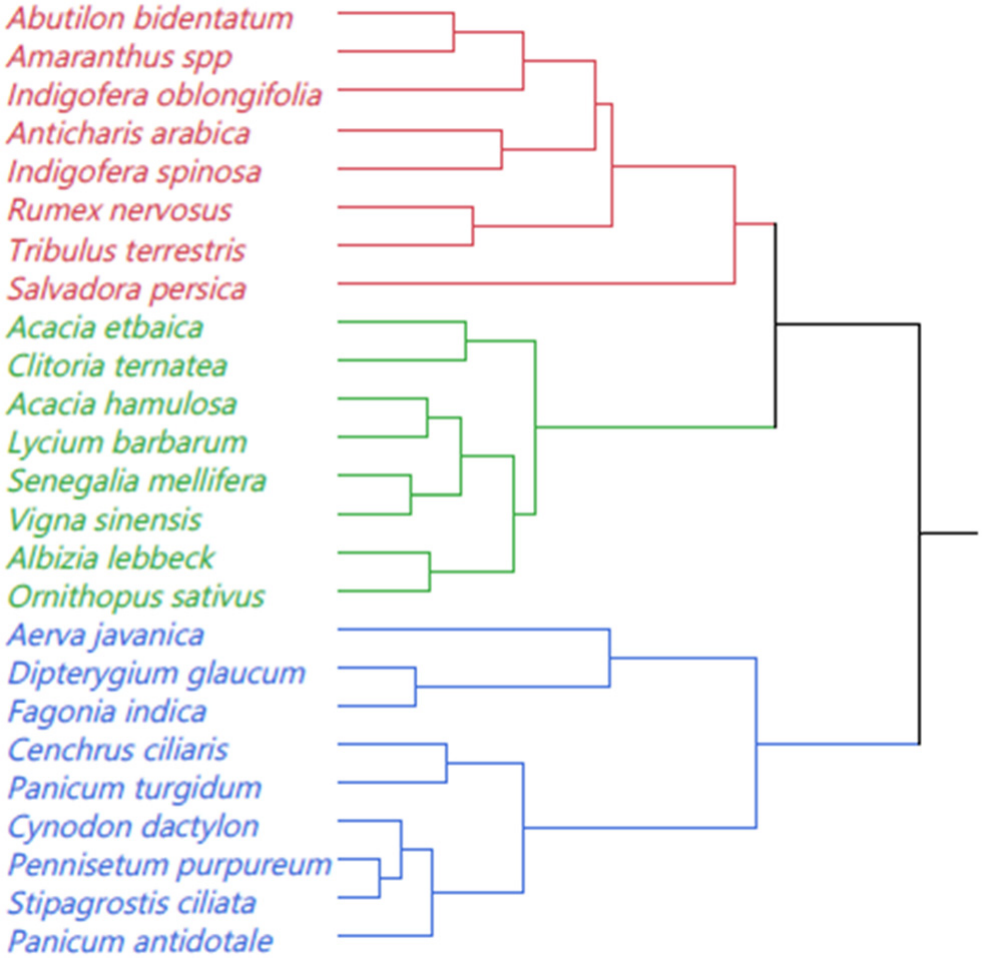
The classification of 25 selected indigenous forage species from southern Yemen based on quality attributes.

The classification of species and their respective quality attributes among rangeland forage species is presented in Figure 2 and Table 3. In Cluster 2, the average crude protein content was approximately twice that of Cluster 3 and 41% higher than Cluster 1. On the other hand, Cluster 3 displayed a crude fiber concentration that was 140% greater than Cluster 1 and 39% higher than Cluster 2. Cluster 1 stood out with significantly higher carbohydrate percentages compared to Clusters 2 and 3, exhibiting a 23 and 29% difference, respectively. Similarly, Cluster 1 had the highest ash content, surpassing Clusters 2 and 3 by 41%. While there were no significant differences in palatability among the three clusters, Cluster 3 exhibited a higher crude fiber content compared to Clusters 1 and 2, showing an increase of 36 and 11.6%, respectively.

**Table 3:** Classification 25 selected indigenous forage species from southern Yemen based on quality attributes

Item	Cluster 1	Cluster 2	Cluster 3
Species	*Abutilon bidentatum*	*Acacia etbaica*	*Aerva javanica*
	*Amaranthus* spp.	*Clitoria ternatea*	*Dipterygium glaucum*
	*Indigofera oblongifolia*	*Acacia hamulosa*	*Fagonia indica*
	*Anticharis arabica*	*Lycium barbarum*	*Cenchrus ciliaris*
	*Indigofera spinosa*	*Senegalia mellifera*	*Panicum turgidum*
	*Rumex nervosus*	*Vigna sinensis*	*Cynodon dactylon*
	*Tribulus terrestris*	*Albizia lebbeck*	*Pennisetum purpureum*
	*Salvadora persica*	*Ornithopus sativus*	*Stipagrostis ciliata*
			*Panicum antidotale*
Moisture (%)	13.5 + 3.02	15.6 + 3.73	11.2 + 1.84
Crude protein (%)	12.5 + 1.24	17.2 + 0.81	8.7 + 0.58
Crude fat (%)	2.4 + 0.24	2.9 + 0.4	3.3 + 0.69
Ash (%)	19 + 2.84	13.3 + 1.02	14 + 1.05
Non-fiber carbohydrate (%)	52.1 + 3.27	42.2 + 1.34	40.2 + 1.82
Crude fiber (%)	14.1 + 1.33	24.5 + 0.99	33.8 + 1.94
Palatability index	4 + 0.33	4.3 + 0.16	4.6 + 0.24

The first cluster comprises eight indigenous forage species, including seven shrubs and one herb (*T. terrestris*), representing various plant families such as Malvaceae, Amaranthaceae, Fabaceae, Scrophulariaceae, Polygonaceae, Zygophyllaceae, and Salvadoraceae. This classification suggests that these species hold promise as highly productive fodder sources. They have the potential to address feed shortages due to their drought resistance and quality [22].

The second cluster consists of eight species, predominantly legumes, which explains the high crude protein content and overall good fodder quality. In contrast, the third cluster includes nine species, mainly grasses, contributing to the high fiber content in this group suggesting their suitability for specific grazing purposes. These findings indicate that rangeland indigenous forage species and their associated fodder quality attributes can be instrumental in identifying promising candidates for rangeland rehabilitation and development.

## 4 Conclusions

Given the limited forage resources in Yemen, there is an urgent need to rehabilitate degraded rangelands and expand the cultivation of fodder species. Our findings demonstrate that all 25 indigenous forage species studied have good potential to support livestock production. Notably, *C. ternatea*, *Lycium barbarum*, *S. mellifera*, *V. sinensis*, *A. lebbeck*, and *Acacia* species show substantial nutritional value and adaptability to the local environment, making them invaluable for addressing fodder scarcity.

This study provides critical information for developing strategies to alleviate feed shortages. This knowledge could inform rangeland management strategies, enabling decision-makers, and pastoral communities to develop sustainable feeding practices that improve livestock production systems and support environmental sustainability.

Incorporating these promising plant species into regular livestock diets could enhance the sustainability and productivity of pastoral systems in southern Yemen. Further field and feed trials are needed to assess the impact of these plant species on livestock performance, as well as their effect on reducing carbon and nitrogen footprints, thus promoting the overall system sustainability.
